# A *Pichia* biosensor for high‐throughput analyses of compounds that can influence mosquito behavior

**DOI:** 10.1002/mbo3.1139

**Published:** 2020-12-02

**Authors:** Julia Nogueira Varela, Vikramaditya G. Yadav

**Affiliations:** ^1^ Department of Chemical and Biological Engineering & School of Biomedical Engineering The University of British Columbia Vancouver BC Canada

**Keywords:** mosquito, odor biosensor, olfactory receptor co‐receptor, public health, repellant, synthetic biology, yeast

## Abstract

Mosquitoes utilize their sense of smell to locate prey and feed on their blood. Repellents interfere with the biochemical cascades that detect odors. Consequently, repellants are highly effective and resource‐efficient alternatives for controlling the spread of mosquito‐borne illnesses. Unfortunately, the discovery of repellents is slow, laborious, and error‐prone. To this end, we have taken a giant stride toward improving the speed and accuracy of repellant discovery by constructing a prototypical whole‐cell biosensor for accurate detection of mosquito behavior‐modifying compounds such as repellants. As a proof‐of‐concept, we genetically engineered *Pichia pastoris* to express the olfactory receptor co‐receptor (Orco) of *Anopheles gambiae* mosquitoes. This transmembrane protein behaves like a cationic channel upon activation by stimulatory odorants. When the engineered *Pichia* cells are cultured in calcium‐containing Hank's buffer, induction of the medium with a stimulatory odorant results in an influx of calcium ions into the cells, and the stimulatory effect is quantifiable using the calcium‐sequestering fluorescent dye, fluo‐4‐acetoxymethyl ester. Moreover, the stimulatory effect can be titrated by adjusting either the concentration of calcium ions in the medium or the level of induction of the stimulatory odorant. Subsequent exposure of the activated *Pichia* cells to a repellant molecule inhibits the stimulatory effect and quenches the fluorescent signal, also in a titratable manner. Significantly, the modular architecture of the biosensor allows easy and efficient expansion of its detection range by co‐expressing Orco with other olfactory receptors. The high‐throughput assay is also compatible with robotic screening infrastructure, and our development represents a paradigm change for the discovery of mosquito repellants.

## Graphical abstract

We have constructed a prototypical whole‐cell biosensor in Pichia pastoris that can be used to study the molecular interactions of the mosquito olfactory receptor co‐receptor (Orco). The sensor can also be expanded through co‐expression of olfactory receptors to study olfactory cascades and identify molecules that can modulate these receptors.

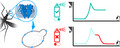

## 1 INTRODUCTION

Mosquito‐borne illnesses such malaria, dengue fever, and infections caused by West Nile and other encephalitic viruses affect over 700 million people across the globe each year (Caraballo & King, 2014; Githeko et al., 2000; Tanser et al., 2003; World Health Organization, 2015). About a million of these perish and most deaths are concentrated in Africa. Worse still, our medical arsenal to guard against these illnesses is declining rapidly (Dondorp et al., 2009), and the burden of these diseases is only going to increase with climate change. New strategies are desperately needed to guard against the spread of mosquito‐borne diseases. Biological vector control has attracted sizable interest in recent years (Benelli et al., 2016) and is typically achieved either by reducing the population of the vector in the wild or making it resistant to the disease (Lambrechts et al., 2015; O’Neill, 2015; Servick, 2016). Both approaches for biological vector control have shown promising results in small‐scale, narrowly focused field trials. Whether this performance can be replicated at larger, more realistic scales remains to be seen, not to mention the uncertainty about their long‐term ecological impacts (David et al., 2013).

The transmission rate of mosquito‐borne diseases can also be reduced by diminishing the number of interactions between mosquitoes and humans. To this end, the use of herbal repellents ranks as one of the oldest techniques to ward off mosquitoes. All female mosquitoes that harbor pathogens utilize their sense of smell to locate and feed on the blood of their targets (Zwiebel & Takken, 2004). Ergo, interfering with this elaborate odor‐sensing mechanism with the aid of attractants or repellents, could offer the greatest protection against the threat of mosquito bites, consequently reducing transmission rates. Saliently, since the use of repellents and attractants does not necessitate lifestyle changes on the part of the users nor require active supervision by medical professionals, they could prove to be highly effective and resource‐efficient alternatives for controlling the spread of mosquito‐borne illnesses (Nguyen et al., 2018; Win et al., 2018).

Chemical interference of mosquito olfaction is an established concept, and the most well‐known repellent, diethyltoluamide (DEET), has been on the market for over seven decades. However, despite its effectiveness, DEET is toxic (Robbins & Cherniack, 1986; Schoenig et al., 1999). Safer and possibly more effective mosquito repellants are preferred, but the current methodology used in the industry for identifying promising candidates—which involves the use of an instrument known as an olfactometer—are not conducive for high‐throughput molecular analysis (DeGennaro et al., 2013; Kröber et al., 2010). As a consequence, only a handful of new repellents have been introduced to the market in decades. The development of a more accurate platform for screening superior mosquito repellents and attractants could turn the tide in the fight to check the transmission rate of mosquito‐borne diseases. To this end, the discovery of mosquito behavior‐modifying compounds could borrow a page from the playbook of the pharmaceutical industry, which has benefitted immensely by “industrializing” drug discovery. Industrialization refers to the acceleration of drug discovery via the miniaturization of assays and reactions, all under robotic control. In the same vein, the development of an accelerated platform for assaying chemical modulators of the mosquito's sense of smell could resuscitate a previously dormant field. Herein, we have laid the foundations for the development of a high‐throughput assay for the detection of mosquito behavior‐modifying compounds by encoding the functional expression of a critical constituent of the olfactory pathway of the malaria‐carrying mosquito *Anopheles gambiae* into the methylotrophic yeast *Pichia pastoris*. The latter is extensively used in the biotechnology industry for the production of proteins and is rated as a model eukaryotic chassis for synthetic biology.

Olfaction in all mosquitoes occurs in their antennae and maxillary palps (Zwiebel & Takken, 2004). These organs are covered in sensory hairs known as sensilla, and each sensillum hosts multiple olfactory receptor neurons (ORNs) that extend into a distinct peripheral dendrite (Leal, 2013). Olfactory transduction commences with the diffusion of odor molecules or odorants through the pores in the sheath of the sensilla. Once inside the sensillum, the odorants then bind to a class of soluble enzymes called odor binding proteins (OBPs) (Bohbot et al., 2007; Wang et al., 2010). Female *A*.* gambiae* mosquitoes, which transmit malaria, express 69 unique OBPs (White et al., 2014). The OBPs subsequently shuttle the odorants to receptor proteins located on the surface of the peripheral dendrites of the ORNs. Mosquitoes and other insects express a variety of odor‐sensing receptor proteins, including olfactory receptors (ORs), ionotropic receptors (IRs), and CO_2_‐sensing gustatory receptors (GRs) (Wicher, 2015). Of these, ORs are the most well‐studied group (Bohbot & Pitts, 2015). They are seven‐transmembrane‐helix proteins that exhibit an inverted topology (Mukunda et al., 2018; Wicher et al., 2008), and female *A*.* gambiae* mosquitoes express 79 ORs (Rinker et al., 2013). Activation of the ORs by the OBP‐odorant complexes, in turn, induces a downstream signaling cascade that activates G‐protein complexes, which subsequently interact with adenylyl cyclase (AC) and phospholipase C (PLC) to produce the secondary messenger molecules, cyclic AMP (cAMP), diacylglycerol (DAG), and inositol 1,4,5‐triphosphate (IP_3_). These messenger molecules then trigger the opening of Ca^2+^ ion channels, thereby generating the transduction currents that are central to the mosquito's sense of smell. In some cases, the odorants themselves can diffuse to the ORs and activate them without the participation of the OBPs (Xiao et al., 2019).

**Figure 1:**
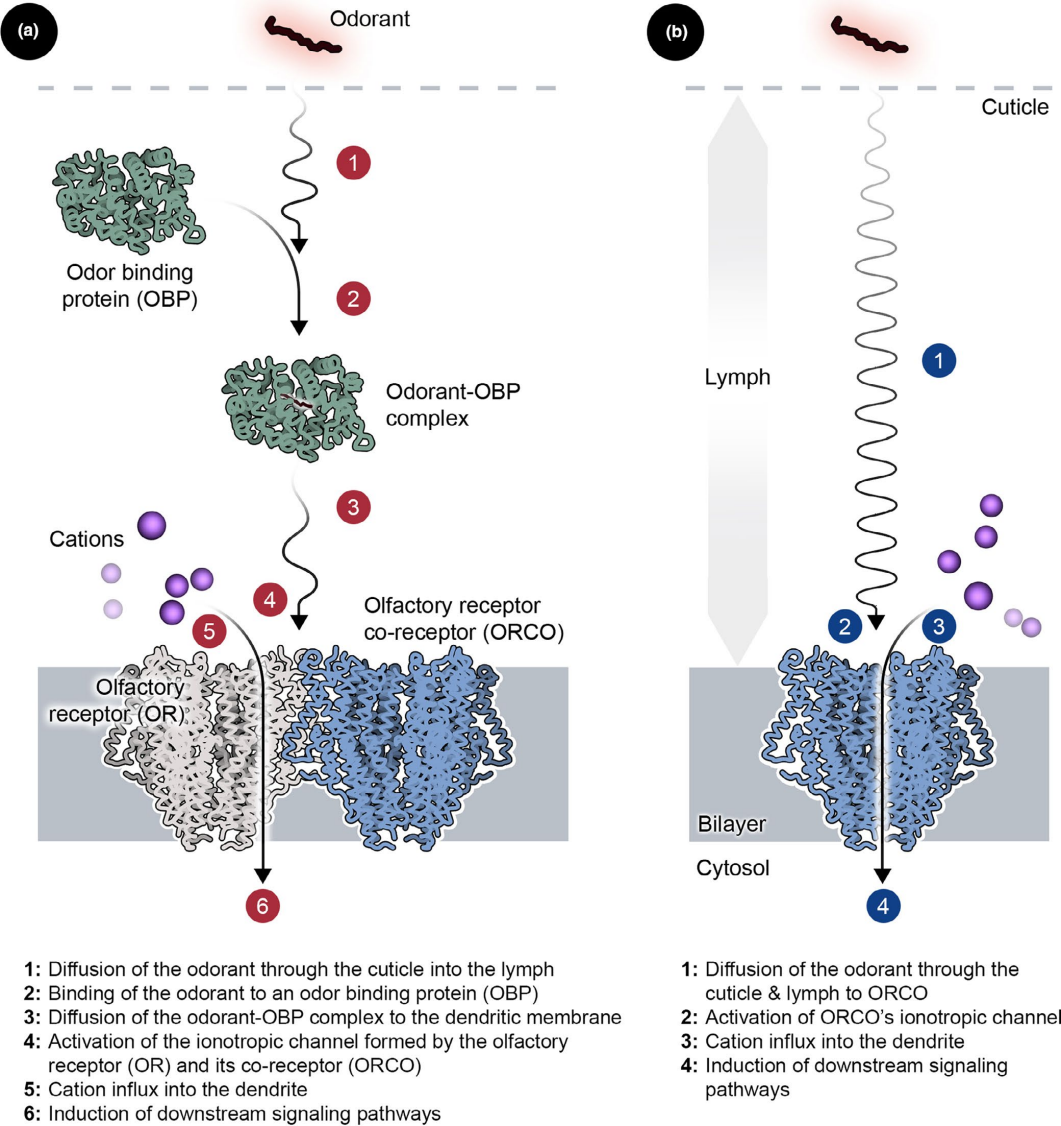
Design of the odor biosensor. (a) The complete odor transduction pathway in mosquitoes involves odor binding proteins (OBPs), olfactory receptors (ORs), and the olfactory receptor co‐receptor (Orco). (b) Previous studies have shown that Orco can function as a cationic channel and individually transduce some olfactory signals. As a consequence, the refactored olfactory transduction pathway was constructed in*P. pastoris*by solely employing Orco.

The OR‐mediated olfactory cascade in mosquitoes also involves another protein called the olfactory receptor co‐receptor (Orco). This protein plays a key role in the signal transduction cascade that originates with the ORs and ends with their cognate G‐protein complexes. It is a homomeric protein that comprises four subunits that are symmetrically arranged around a central channel (Butterwick et al., 2018). Interestingly, it has been suggested that Orco itself can function as a cationic channel upon activation by some OBP‐odorant complexes and odorants (Jones et al., 2011). Not only does this property of Orco make it a promising target for repellant design (Leal, 2013), but the comparable structural complexity between Orco with other ORs makes the former an excellent candidate for assessing the possibility of expressing olfactory proteins in microbial hosts for use as whole‐cell biosensors of mosquito behavior‐modifying compounds. Functional expression and modulation of olfactory proteins by behavior‐modifying could be rapidly probed by measuring cation influx into the microbial host (Figure 1), thereby offering a more robust, versatile, and customizable platform for high‐throughput screening of mosquito behavior‐modifying compounds.

Nevertheless, mosquito olfactory proteins have only ever been functionally expressed in HEK293 cells (Rinker et al., 2012), Sf9 cells (Jordan & Challiss, 2011; Kiely et al., 2007), and *Xenopus* oocytes (Misawa et al., 2010). These cell lines are cumbersome and expensive to maintain and are largely incompatible with high‐throughput screening. The successful expression of mosquito olfactory proteins in a facilitatively transformable and modularizable microbial chassis would represent a significant advance and lay strong foundations for the eventual development of whole‐cell biosensors that recapitulate the more complex olfactory cascades. To this end, we genetically engineered *P*.* pastoris* to express the Orco protein of female *A*.* gambiae* mosquitoes. The use of this *Pichia* biosensor for evaluation of the stimulatory or repellatory commences with incubation of the cells in a buffered solution that contains the calcium‐sequestering dye, fluo‐4‐acetoxymethyl ester (abbreviated as fluo‐4‐AM), in a standard multi‐well plate. The calcium‐bound form of the dye is highly fluorescent, whereas the fluorescence emitted by its free form is barely detectable. Calcium‐bound fluo‐4‐AM has an excitation and emission wavelength of 485 and 520 nm, respectively. After allowing sufficient time for the dye to infuse the cells, we transfer the cells to a multi‐well plate in a calcium‐containing medium and later inject odorants into the solution. Stimulatory odorants activate Orco and trigger the influx of calcium ions from the solution into the cells, and the increase in intracellular calcium concentration generates a fluorescent signal that can be titrated by adjusting either the concentration of calcium ions or odorant in the solution. If repellatory or inert odorants are added to the solution instead of a stimulatory molecule, they will not change the intracellular concentration of calcium ions. However, a repellant can be differentiated from an inert odorant by preceding its addition to the medium with the introduction of a known stimulatory odorant. The repellant will dampen or plateau the fluorescent signal, whereas an inert molecule that does not interact with Orco will not affect the fluorescence emitted by the cells.

We assessed the performance of the *Pichia* biosensor to quantify the stimulatory or repellatory effect of odorants that have been studied previously in exceptionally complicated investigative models such as empty neurons of *Drosophila melanogaster* (Fleischer et al., 2018). Specifically, we exposed the biosensor to oct‐1‐en‐3‐ol, a known mosquito attractant that is present in human sweat but does not activate Orco (Meijerink et al., 2001); 2‐(4‐ethyl‐5‐(pyridin‐3‐yl)‐4H‐1,2,4‐triazol‐3‐ylthio)‐N‐(4‐ethylphenyl)acetamide or VUAA1, one of the strongest activators of Orco reported in the literature (Rinker et al., 2012), and citronella oil, whose effect on Orco remains poorly understood (Nguyen et al., 2018). Not only does the sensitivity (based on EC50 values) and specificity of the *Pichia* biosensor compare favorably to previously reported systems, but it is manifold faster to construct and deploy. Moreover, its modular architecture allows easy and efficient expansion of its detection range by co‐expression of Orco with any of the 79 ORs expressed by female *A*.* gambiae* mosquitoes. This simple biosensor can form the basis of a high‐throughput, high‐resolution platform for detecting chemical modulators of the mosquito's sense of smell that, perhaps most significantly, is cheap, modular, and compatible with robotic screening infrastructure in place in the pharmaceutical industry. Moreover, it could also be easily adapted to study pollination and insect aromachology.

## 2 MATERIALS AND METHODS

### 2.1 Molecular cloning and transformation of *P*.* pastoris*

We used the *P*.* pastoris* GS115 as the chassis for all experiments in this study. *P*.* pastoris* exhibits a significantly shorter doubling time, is easier to work with, and its toolkit for molecular cloning (Ahmad et al., 2014; Byrne, 2015; Higgins, 2004; Krettler et al., 2013) is much more developed than competing platforms such as *Xenopus* oocytes (Fleischer et al., 2018), *Drosophila* empty neurons (Fleischer et al., 2018), HEK 293 (Cervera et al., 2011), and Sf9 cells (Betenbaugh et al., 1991). Moreover, *P*.* pastoris* has been used previously to express heterologous membrane proteins (Fukutani et al., 2012, 2015; Fukutani, et al., 2012; Radhika et al., 2007). The amino acid sequence of Orco was retrieved from the UniProt database and reverse‐translated to acquire its cDNA sequence. The latter was then codon‐optimized for expression in *P*.* pastoris* and synthesized through a commercial service (GenScript). The *Pichia* cells were transformed using the pPICZA plasmid. The coding sequences on the plasmid are under the transcriptional control of the AOX1 promoter, which is inducible in a titratable manner with methanol. The plasmid also bears a zeocin (phleomycin D1) selection marker, and its multi‐cloning site (MCS) is configured to allow the inclusion of C‐terminal c‐myc and polyhistidine (6xHis) tags. The *P*.* pastoris* GS115 strain and the pPICZA plasmid were generously donated to us by Prof. Steven Hallam (Department of Microbiology & Immunology, The University of British Columbia).

All sub‐cloning was performed in *E*.* coli* DH5α and commenced with the digestion of the pPICZA plasmid and PCR product of Orco cDNA using EcoRI and NotI and their subsequent ligation using T4 DNA ligase to generate the pOrco plasmid. We subsequently linearized the pOrco plasmid using the SacI enzyme. All restriction endonucleases used in the study were of the high‐fidelity form, and all enzymes were purchased from New England Biolabs. The *Pichia* cells were then made electrocompetent and transformed with the previously linearized pOrco plasmid via electroporation (Krettler et al., 2013). The transformed cells were plated onto YPD plates (1% yeast extract, 2% peptone, 2% dextrose, and 2% agar) containing 100 µg/ml of zeocin and incubated in a darkened incubator at 30°C for 2–3 days to yield colonies that are sufficiently large to be observable with the naked eye. Transformation of *P*.* pastoris* GS115 was confirmed through colony PCR, and the resultant clone is labeled as PP‐Orco.

### 2.2 Culturing conditions for expression of Orco

We arbitrarily selected three colonies for further testing. The colonies were transferred to 5 ml of YPD medium (1% yeast extract, 2% peptone, and 2% dextrose) and cultured overnight at 30°C under constant agitation at 200 rpm. Unless otherwise noted, all liquid culturing media described hereinafter also contained 100 µg/ml of zeocin. We then inoculated 0.1 ml of these cultures in triplicate in 30 ml of BMGY medium (1% yeast extract, 2% peptone, 1% glycerol, 400 μg/l biotin, 0.1 M potassium phosphate, and pH 6.0) and propagated the cultures in 250 ml baffled flasks for 24 h. Next, we centrifuged the cultures at 800 rcf for 3 min and resuspended the cell pellets in 30 ml of BMMY media (1% yeast extract, 2% peptone, 0.1% methanol, 400 μg/l biotin, 0.1 M potassium phosphate, and pH 6.0). The approximate average optical densities of the cultures at 600 nm (OD_600_) at this point was 1. The BMMY cultures were then propagated at 30°C for 48 h under constant agitation at 200 rpm. Methanol in the culture medium was topped up to 0.01% every 18 h. At the end of culturing, we centrifuged the cultures at 1,800 rcf and 4°C and subsequently harvested the cells and stored them at −80°C before Western blotting. We also tested the BMMY medium containing 0.01% methanol to evaluate the impact of the concentration of the inducer on the expression of Orco.

### 2.3 Processing of harvested *Pichia* cells for Western blotting

The previously frozen cell pellets were resuspended in 10 ml of breaking buffer (50 mM potassium phosphate, 100 mM NaCl, 1 mM EDTA, 5% glycerol, and 100 mM PMSF) and centrifuged for 5 min at 2,500 rcf and 4°C. The supernatant was collected and centrifuged for another 15 min at 35,000 rcf and 4°C. The supernatant that emerges from the second centrifugation was centrifuged once more for 1 h at 100,000 rcf and 4°C. The resulting pellet, which contains the membrane fraction, was resuspended in a membrane buffer solution (50 mM Tris, 120 mM NaCl, 2 mM EDTA, 10% glycerol, and pH 8) and used directly for Western blotting. We also diluted 25 μl of the supernatant produced in the second centrifugation step and diluted it with 25 μl of 2x Laemmli buffer for analysis using Western blotting. This sample represents the whole‐cell fraction.

### 2.4 Western blotting

The membrane and whole‐cell fractions of the PP‐Orco cultures were resolved using protein gel electrophoresis on pre‐cast Mini‐PROTEAN TGX gels. Electrophoresis was performed for 1 h at 90 V. The gels were then transferred to an Immun‐Blot PVDF membrane in a blotting cell that was filled with ice‐cold transfer buffer (25 mM Tris, 1.92 M glycine, 20% methanol, and pH 8.5). The transfer took place over 1 h at 100 V, and the membrane was then blocked for 1 h under constant, gentle shaking at room temperature using a mixture of TBST solution (137 mM NaCl, 19 mM Tri‐Base, and 1% Tween‐20) and 5% skim milk. The blocked membrane was subsequently washed with the TBST solution and incubated overnight with the primary antibody for the c‐myc tag at 4°C under constant shaking, which was followed by a shorter incubation of 1 h with a suspension of the HRP conjugate of the goat anti‐mouse IgG secondary antibody in 5% skim milk at room temperature. The membranes were imaged on a Clarity ECL substrate in a ChemiDoc MP Imager.

### 2.5 Assessment of dye permeation into *Pichia* cells

We used confocal microscopy to assess the permeation of fluo‐4‐AM into the *Pichia* cells. Overnight cultures of *P*.* pastoris* GS115 and PP‐Orco in 5 ml of YPD were centrifuged at 2,000 rcf for 15 min at room temperature and resuspended in Hank's buffer with 1 mM Ca^2+^ to achieve a final optical density of 0.4. We then added fluo‐4‐AM to the cell suspensions to achieve a final concentration of 2.5 μM and incubated the solutions for 45 min at 37°C and in a darkened chamber. Next, the cell suspensions were centrifuged at 2,000 rcf for 15 min at room temperature and the resultant pellet was resuspended in fresh PBS. We repeated the centrifugation and resuspension in PBS two more times. The cells were imaged using an Olympus FV‐1000 laser‐scanning confocal microscope under 60× magnification and at excitation and emission wavelengths of 488 and 505 nm, respectively.

### 2.6 Assay for assessment of the *Pichia* biosensor

The PP‐Orco cultures were initially propagated in 30 ml of BMGY medium for 24 h. When the OD_600_ readings of the cultures reached 1, they were centrifuged for 5 min at 2000 rcf and room temperature. The cell pellets were resuspended in 30 ml BMMY media containing 0.1% methanol and propagated for 48 h. The cultures were later centrifuged for 5 min at 5000 rcf, and room temperature and the resultant pellets were resuspended in a 1:1 volumetric mixture of BMMY media and PBS to a final OD_600_ reading of 0.4. Fluo‐4‐AM was added to the solutions to a concentration of 2.5 μM, and the cells were incubated with the dye for 45 min at 37°C in a darkened chamber. The cell suspensions were later centrifuged at 2000 rcf for 15 min at room temperature, and the resultant pellet was resuspended in fresh PBS. We repeated the centrifugation and resuspension in PBS two more times. After the final PBS wash, the cells were resuspended in Hank's buffer to achieve an OD_600_ value of 0.4, and 200 μl of this suspension was pipetted in each well of a 96‐well plate. Hank's buffer is a calcium‐containing medium. We evaluated the biosensor in Hank's buffer containing 1 mM and 5 mM of Ca^2+^.

After allowing the basal fluorescence in each well to equilibrate, which takes approximately 6 min, we injected the odorants into each well. We tested VUAA1 (0.125, 0.25, 0.50, 1, 1.50, and 2 mM), citronella oil (1:15, 1:7, 1:3, and 1:1 volumetric dilution in 1 μl solution with DMSO), and oct‐1‐en‐3‐ol (1:7, 1:3 and 1:1 volumetric dilution in 1 μl solution with DMSO) individually. The fluorescence emitted by each well was recorded over 6 min using excitation and emission wavelengths of 485 and 520 nm, respectively. We also assessed the behavior of the sensor when it is sequentially exposed to citronella oil followed by VUAA1. We added VUAA1 to the well 2 min after the addition of citronella oil. Normalization of the fluorescence recordings was performed as follows:

(1) $$F_{\text{sample}}‐F_{\text{sample}}^{o}=\Delta F$$

(2) $$F_{\text{blank}}‐F_{\text{blank}}^{o}=\Delta F_{\text{blank}}$$

(3) $$\frac{\Delta F}{F_{\text{sample}}^{o}}‐\frac{\Delta F_{\text{blank}}}{F_{\text{blank}}^{o}}=F$$

*F*_sample_ and *F*_blank_ are the fluorescence recordings for the sample and control wells, respectively, at a given time point, whereas $F_{\text{sample}}^{o}$ and $F_{\text{blank}}^{o}$ are the fluorescence recordings for the sample and control wells at the initial time point. Hereinafter, fluorescence refers to the normalized fluorescence, *F*. We analyzed three technical and two biological replicates for each assay condition, and statistical significance was assessed by performing a one‐way ANOVA paired test with a Tukey post hoc test on the highest value of the normalized fluorescence for each sample.

## 3 RESULTS

### 3.1 *P*.* pastoris* successfully expresses Orco

**Figure 2:**
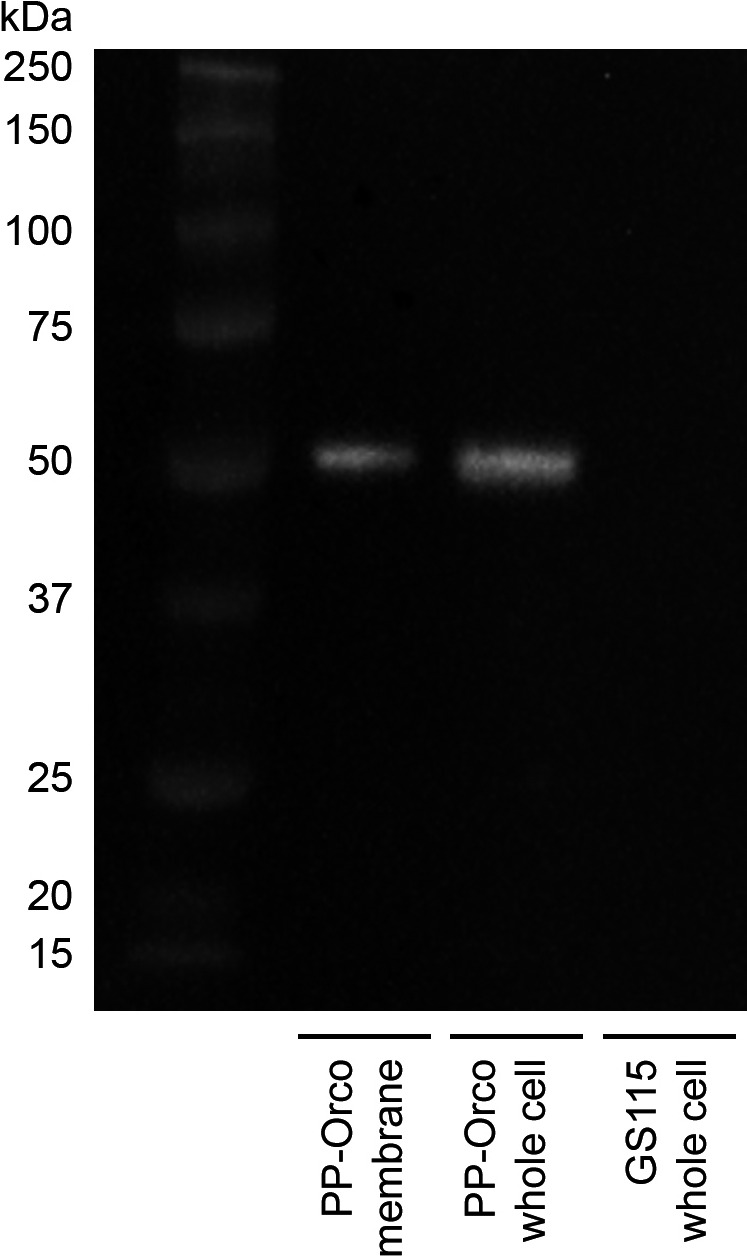
Western blot analysis of*P*.* pastoris*cultures induced with 0.1% methanol confirms expression and correct localization of Orco in the membrane of*P*.* pastoris*. An inverted image of the blot has been provided for visual clarity. Uninduced cultures do not generate a detectable signal, which suggests that the AOX1 promoter is tightly regulated methanol (data not shown). This observation is consistent with previous reports about the promoter (Chang et al.,^2018^).

We tested two concentrations of methanol in the BMMY medium, 0.01%, and 0.1%. Although Orco is expressed by the cells at both induction levels, the expression is markedly higher at 0.1% methanol. Additionally, we did not observe any expression after 24 h of culturing, which suggests that expression and localization of Orco within the membranes of *P*.* pastoris* cells occurs between 24 and 48 h. As a result, we used 0.1% methanol in BMMY medium for all subsequent experiments. Western blotting confirms that *P*.* pastoris* expresses and localizes Orco to its cellular membrane (Figure 2). The unmodified form of Orco has a molecular weight of 54 kDa whereas its glycosylated form weighs approximately 56.5 kDa. The blot image confirms the presence of a protein within the appropriate size window in the PP‐Orco samples. We did not quantify the number of copies of the Orco gene that were incorporated by the Pichia cells and it is highly likely that multiple copies may have been integrated into the genome. However, we have eliminated this ambiguity by screening multiple colonies for the expression of Orco. All subsequent results and their accompanying statistics are calculated using technical replicates for the colony with the strongest bands in the Western blot. Although we did not mutagenize the protein at its sole glycosylation site (N167) to determine if the engineered cells are post‐translationally modifying the protein at the correct location, functional studies presented subsequently confirm that fact. Crucially, no bands were observed in the whole‐cell fraction of *P*.* pastoris* GS115. Moreover, the band intensities for the two PP‐Orco fractions suggest that a majority of the heterologously expressed proteins correctly localize to the membrane. Incidentally, we had previously attempted to construct a version of the biosensor in *Escherichia coli*. We observed that the prokaryotic host was unable to localize Orco to its periplasmic membrane and instead formed inclusion bodies despite co‐expression of chaperones such as DnaK and AAA proteinases such as FtsH (Link et al., 2008).

### 3.2 Fluo‐4‐AM permeates into *P*.* pastoris*

**Figure 3:**
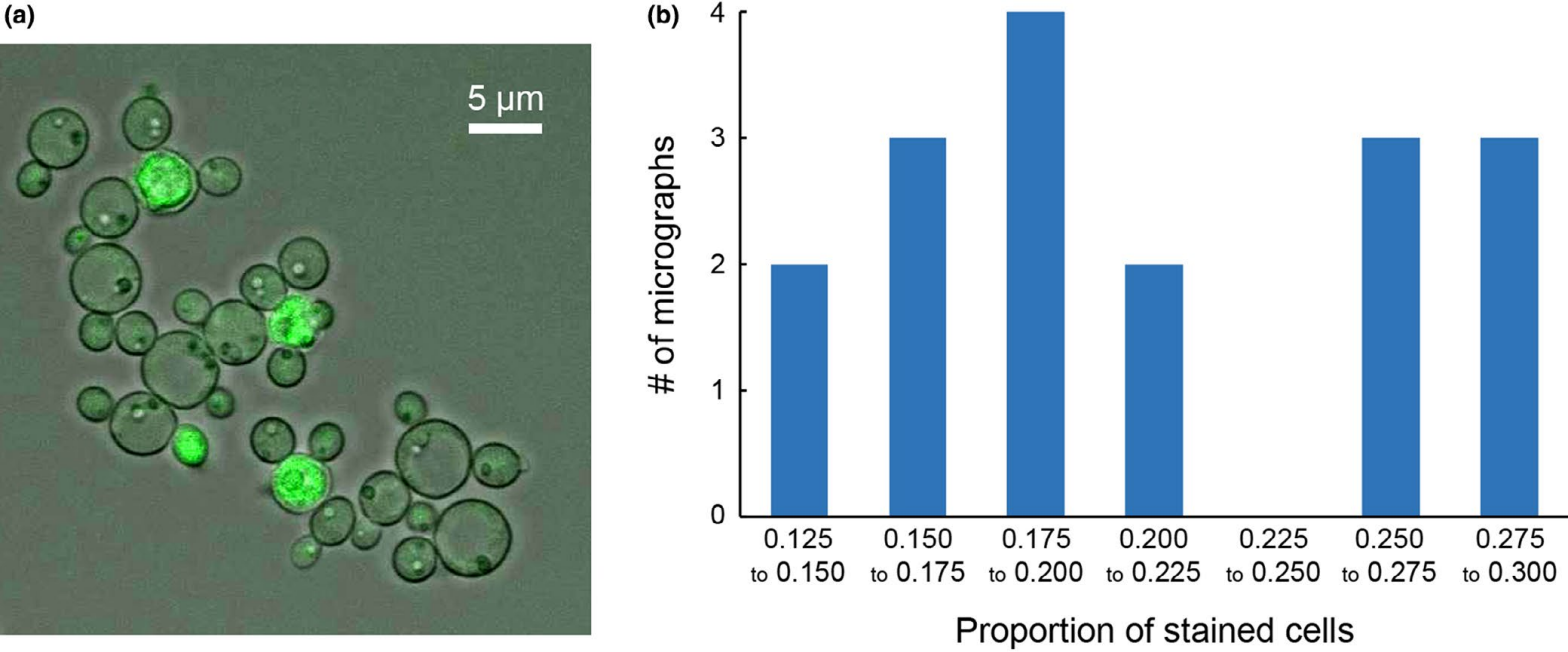
Confocal microscopy and permeation statistics. (a) The confocal micrograph confirms the permeation of fluo‐4‐AM into*P*.* pastoris*. The cells were imaged using an Olympus FV‐1000 laser‐scanning confocal microscope under 60x magnification and at excitation and emission wavelengths of 488 and 505 nm, respectively. (b) The permeation is non‐uniform. However, 45 min of incubation are sufficient to stain about 21% of the population and generate a detectable signal.

The permeation of fluo‐4‐AM into the PP‐Orco cells is arguably the most important step in the implementation of the odor assay and confocal microscopy confirmed that the dye permeates into approximately 21 ± 5% of *Pichia* cells within 45 min (Figure 3). Additionally, we observed that the proportion of cells into which the dye permeates is directly proportional to the incubation time. However, dye permeation is non‐uniform. We speculate that since the cells were incubated with the dye in Hank's buffer, fluo‐4‐AM diffuses into the cell as a complex with Ca^2+^, which lowers its diffusivity and total flux into the cells. Moreover, the non‐uniformity in the distribution of fluorescent cells can also be attributed to differences in the stoichiometric ratio between the dye and Ca^2+^ ions in the complexes. We do not anticipate these phenomena to occur in the assay since incubation of the cells with the dye occurs in PBS in those tests. In light of this conclusion and the fact that we still observe a detectable signal in the assays despite the dye permeating into a little more than a fifth of the population of cells, we decided against optimizing the dye incubation time.

### 3.3 Orco functions like a TRP channel in *P*.* pastoris*

**Figure 4:**
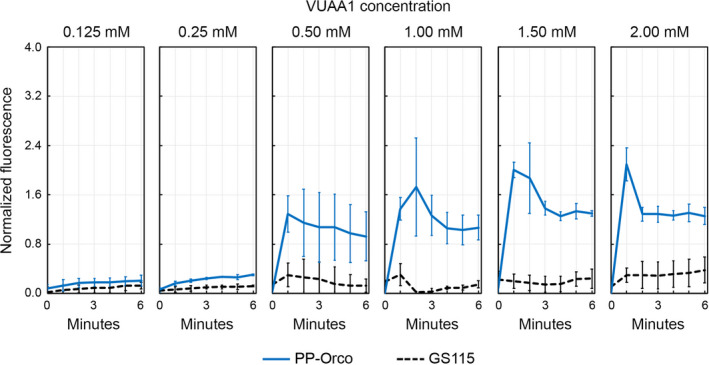
Functional testing of the biosensor in Hank's buffer containing 1 mM Ca^2+^confirms dose‐dependent activation of Orco by VUAA1. The response is analogous to membrane fluctuations observed following activation of a TRP cationic channel. The p‐values for the difference in normalized fluorescence between PP‐Orco and GS115 strains were lower than 0.01 for the 0.5, 1, and 2 mM runs and 0.001 when the concentration of VUAA1 was 1.5 mM.

**Figure 5:**
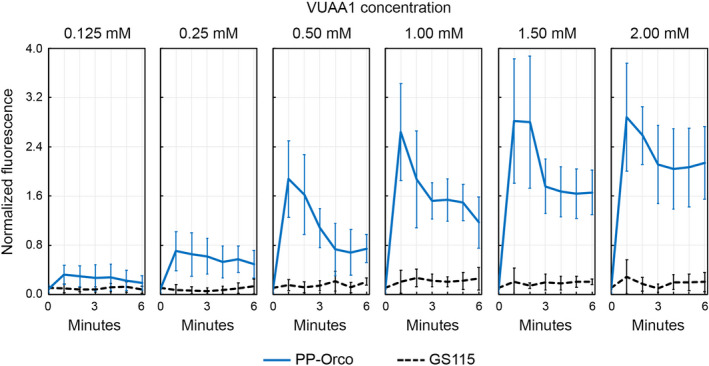
Functional testing of the biosensor in Hank's buffer containing 5 mM Ca^2+^reveals that the signal can be amplified by increasing the concentration of calcium ions in the extracellular medium. The p‐value for the difference in normalized fluorescence between PP‐Orco and the control is < 0.01 when the VUAA1 concentration is 0.125 mM. The corresponding p‐values for the remaining VUAA1 concentrations are below 0.001.

VUAA1 is one of the most potent agonists of Orco and was once a prime candidate for use as a repellant since it was reported to overstimulate mosquitoes to the point of confusion (Rinker et al., 2012). Unfortunately, VUAA1 also exhibits exceptionally low volatility, which presents significant challenges to its use as a repellant. Nevertheless, it is an excellent ligand to test the behavior of the *Pichia* biosensor in liquid cultures. We exposed the PP‐Orco cells to increasing concentrations of VUAA1 in the presence of 1 mM (Figure 4) and 5 mM of Ca^2+^ (Figure 5) in Hank's buffer. VUAA1 was added to the PP‐Orco cultures by suitably diluting a stock solution of the molecule in DMSO. In the case of PP‐Orco cultures that are exposed to 1 mM of extracellular Ca^2+^, exposure to 0.125 mM and 0.25 mM of VUAA1 did not produce a statistically significant response compared to unmodified *P*.* pastoris* GS115 cells but concentrations over 0.50 mM generate a distinct, statistically significant response compared to the control. The p‐values for the difference in normalized fluorescence between PP‐Orco and GS115 strains were lower than 0.01 for the 0.5, 1 and 2 mM runs and 0.001 when the concentration of VUAA1 was 1.5 mM. The normalized fluorescence emitted by these cultures reaches a maximum of 1 minute after the compound has been injected into the medium. The normalized fluorescence then gradually drops over the next 2–3 min, after which it equilibrates for the remainder of the run. This response closely mirrors the fluctuations in membrane potentials that are induced by followed activation of transient receptor potential (TRP) channels such as TRPA1 in *D*.* melanogaster* and *A*.* gambiae* (Kwon et al., 2010; Salgado, 2017; Venkatachalam & Montell, 2007). TRP channels are ion channels that transduce a range of stimuli, including heat, light, taste, pain, and pressure; and the functional similarity between a seven‐transmembrane‐helix protein such as Orco and six‐transmembrane‐helix TRP channels is notable. The sensitivity and signal‐to‐noise ratio of the biosensor was uniformly higher when it was exposed to 5 mM Ca^2+^ and the TRP‐like response was noticeable at all concentrations of VUAA1 that were tested. This observation confirms that the biosensor's readout can be titrated by changing the concentration of Ca^2^ in the extracellular medium. A statistically significant difference in normalized fluorescence between PP‐Orco and the control is measurable at concentrations of VUAA1 as low as 0.125 mM. The *p*‐value for this statistic is < 0.01, whereas the p‐values for the remaining VUAA1 concentrations are below 0.001.

### 3.4 Orco expressed by *P*.* pastoris* has comparable sensitivity to a mammalian system

**Figure 6:**
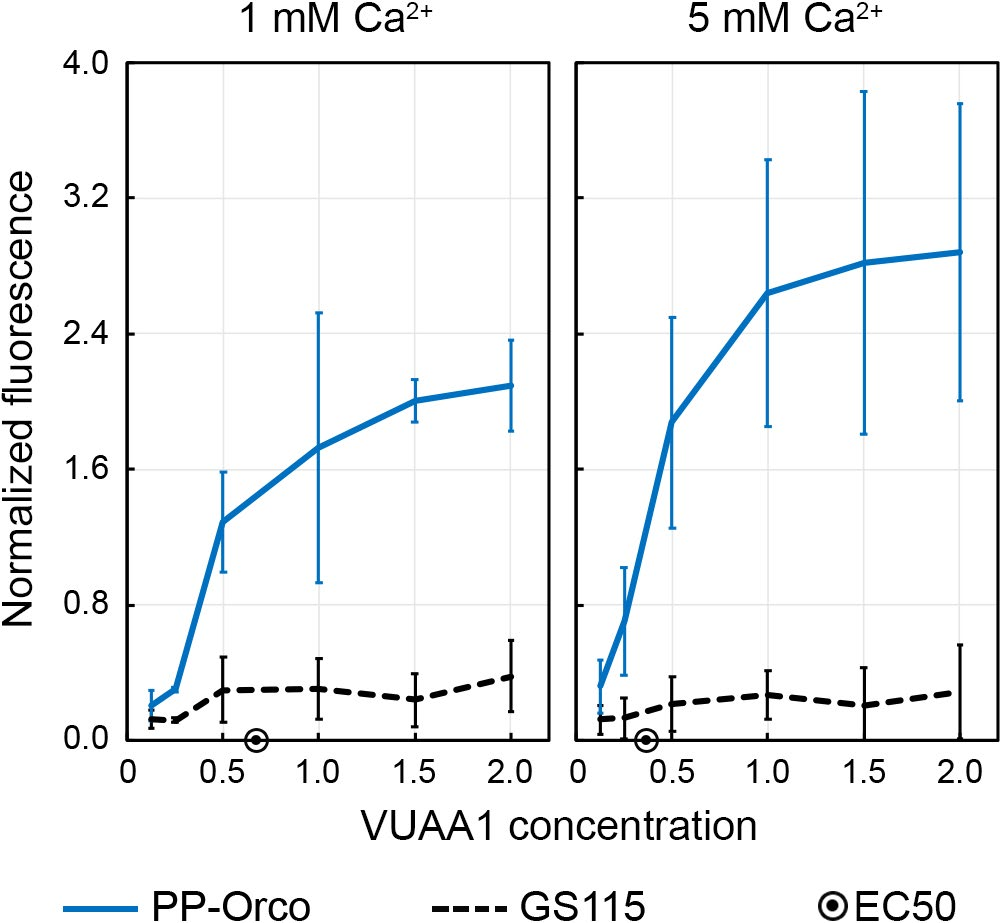
EC50 for the interaction between VUAA1 and Orco is estimated to be 0.85 and 0.41 mM for extracellular calcium concentrations of 1 and 5 mM, respectively. We plotted the highest value of the normalized fluorescence in Figures4and5and its corresponding standard deviation against the concentration of VUAA1 that was used in the reaction. The data were then processed using GraphPad Prism 7 to determine the EC50 values.

The EC50 of a ligand is its concentration that induces a half‐maximal response by its cognate receptor. We plotted the highest value of the normalized fluorescence estimated in each of the aforementioned runs as a function of the concentration of VUAA1 in the sample and determined the EC50 for the activation of Orco by VUAA1 to be 0.85 mM and 0.41 mM for extracellular calcium concentrations of 1 mM and 5 mM, respectively (Figure 6). Although EC50 values of VUAA1 have not been explicitly determined in *A*.* gambiae*, they have been calculated for genetically engineered HEK293 cells and *Xenopus* oocytes that express Orco, albeit using electrophysiological measurements (Rinker et al., 2012). Not only is the *Pichia* system equally sensitive as the HEK293 system, but it offers other advantages such as greater modularity, simpler optimization, and easier deployment in an assay.

### 3.5 DMSO does not interfere with Orco in the assay

**Figure 7:**
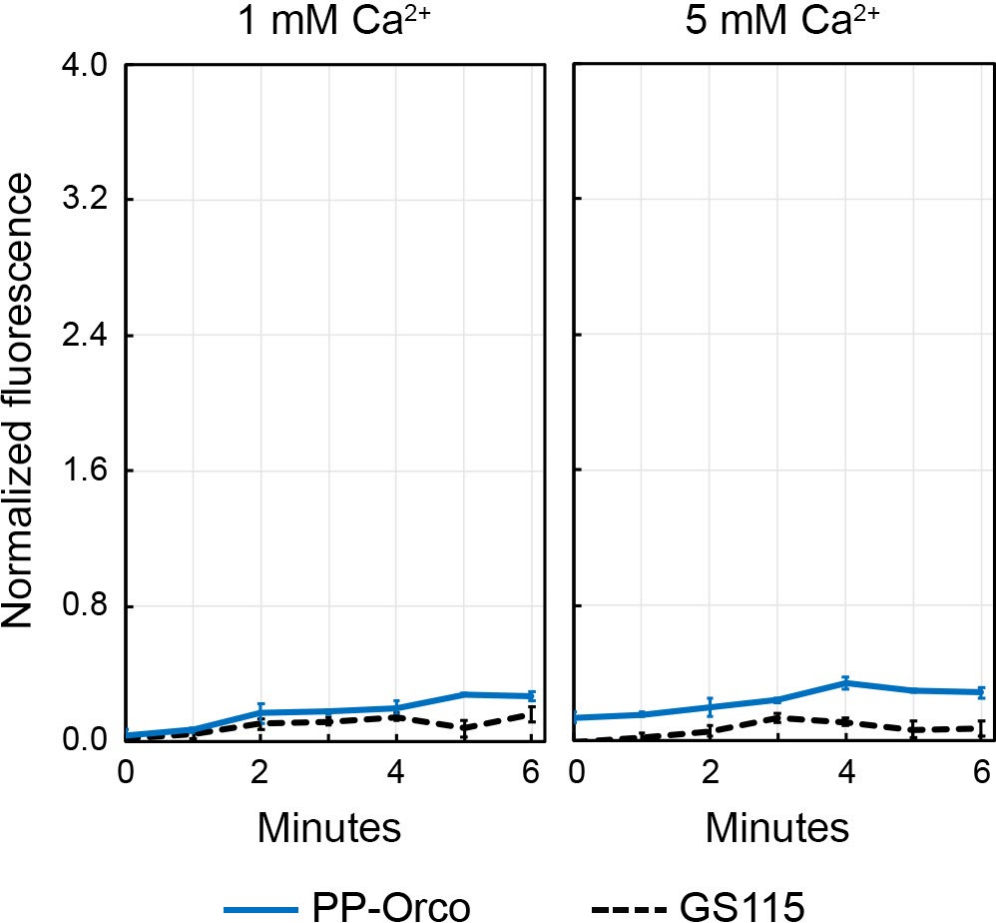
DMSO does not interact with Orco and can be used to solubilize hydrophobic odorants in the assay buffer.

The highest concentration of VUAA1 to which the PP‐Orco cells were exposed was 2 mM and the assay buffer that was used to evaluate this condition was prepared by injecting roughly 1 µl of a 400 mM stock solution of the molecule in DMSO into 200 µl of Hank's buffer. Since DMSO is known to impact cells in myriad ways and could potentially interfere with the activity of Orco, we also assessed the behavior of PP‐Orco cells in Hank's buffer that was injected with 1 µl of pure DMSO. This volume is nearly identical to the volume of DMSO used in the 2 mM VUAA1 test, and we monitored the cells in buffers containing 1 mM and 5 mM Ca^2+^ (Figure 7). The normalized fluorescence emitted by the PP‐Orco cultures is slightly higher than the signal produced by cultures of *P*. pastoris GS115 but this difference is not statistically significant. Moreover, the trend that is observed does not mirror that of PP‐Orco cultures exposed to VUAA1. There is also no significant difference between the normalized fluorescence emitted by PP‐Orco in media containing 1 and 5 mM of extracellular calcium. These observations, in conjunction with data recorded in the subsequent experiment using citronella oil, verify that DMSO does not impact Orco in any capacity at the volumes that were considered in this study.

### 3.6 A component in citronella oil could be modulating Orco

**Figure 8:**
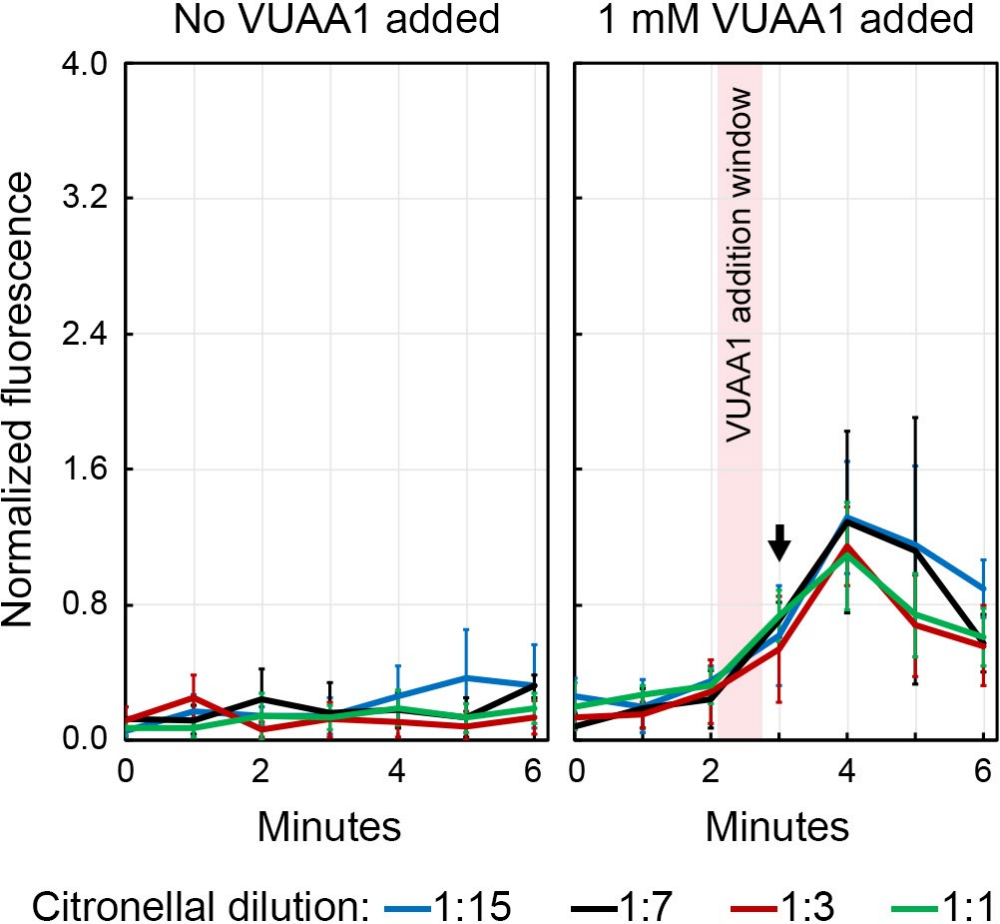
Citronellal or a minor constituent of citronella oil interferes with Orco. The ligand is either a negative allosteric modulator of Orco or an antagonist that is weakly competitive with VUAA1. The dilutions correspond to volumetric ratios of citronella oil and DMSO in an injection of 1 µl into the assay buffer.

Citronella oil is an essential oil that is extracted from the leaves and stem of lemongrass and is widely used around the world as an insect repellant (Nguyen et al., 2018). The active repellatory agent in citronella oil is the monoterpenoid molecule, citronellal. While the interaction between citronellal and Orco has been studied previously in *D*.* melanogaster* (Kwon et al., 2010), its effect on Orco in *A*.* gambiae* is unclear. We employed the PP‐Orco biosensor to systematically probe the interaction between citronella oil and Orco in Hank's buffer containing 5 mM of Ca^2+^. Since citronella oil is highly volatile and largely insoluble in aqueous solutions, which precludes accurate molarity measurements, we pipetted 1 µl of 1:1, 1:3, 1:7, and 1:15 mixtures (volume basis) of citronella oil and DMSO directly into the assay buffer in each well and recorded the fluorescence over 6 min. The citronella oil comprises 93% citronellal. We did not record a statistically significant difference between the fluorescence emissions by the samples (Figure 8). However, the addition of VUAA1 to a final concentration of 1 mM in the medium approximately 2 min into the run activated Orco and generated a TRP‐like response. Importantly, the activation was proportional to how diluted citronellal oil was in the solution. Besides, reversing the order of the addition of VUAA1 and citronella oil did not dampen the fluorescence. These observations raise the possibility that citronellal or another constituent of citronella oil is either a negative allosteric modulator of Orco or an antagonist that is weakly competitive with VUAA1. We did not investigate this phenomenon since it was not the focus of the current study but it warrants a detailed examination in a future study.

### 3.7 Orco is not activated by oct‐1‐en‐3‐ol in human sweat

**Figure 9:**
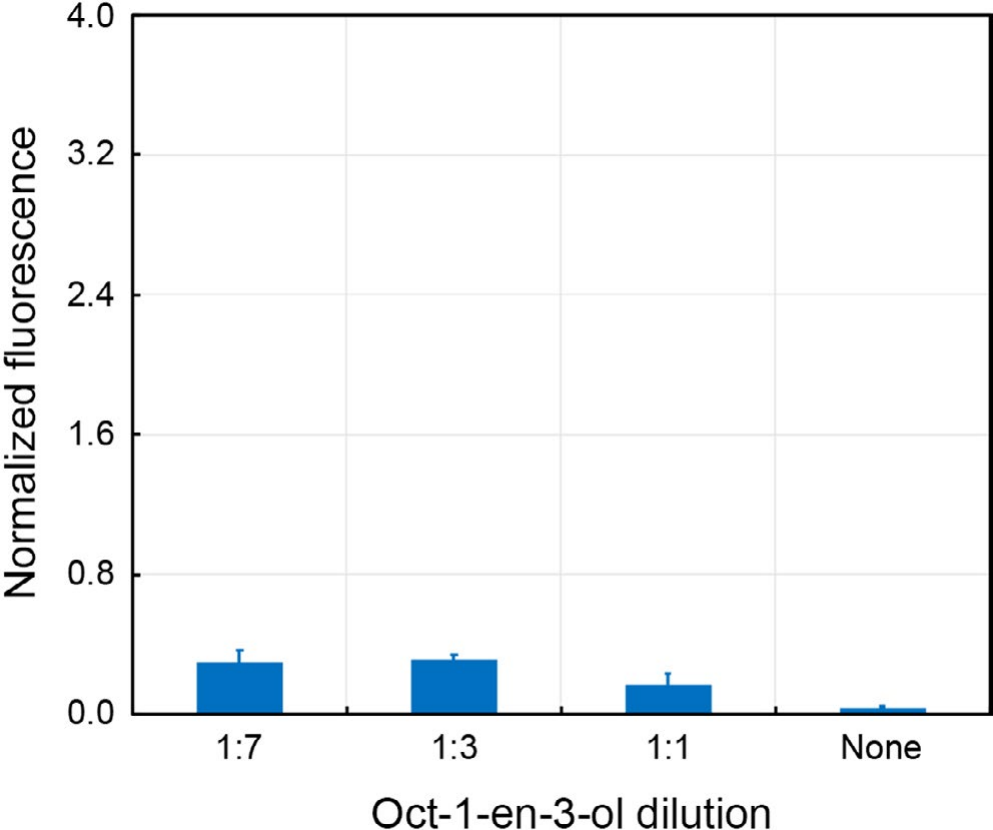
The*Pichia*biosensor does not respond to oct‐1‐en‐3‐ol, which corroborates previous reports that Orco is not activated by the odorant. The dilutions correspond to volumetric ratios of oct‐1‐en‐3‐ol and Hank's buffer containing 5 mM Ca^2+^in an injection of 1 µl into the assay buffer. “None” refers to no dilution.

Female *A*.* gambiae* mosquitoes are primarily attracted by oct‐1‐en‐3‐ol in human sweat and a previous study by another group revealed that the molecule does not interact with Orco but instead activates other ORs (Xu et al., 2015). It has even been suggested that DEET repels mosquitoes by reducing the volatility of oct‐1‐en‐3‐ol rather than inhibiting any interactions with ORs (Afify et al., 2019; Syed & Leal, 2008). We investigated the interaction between oct‐1‐en‐3‐ol and Orco by injecting 1 µl of the undiluted molecule and 1:1, 1:3, and 1:7 mixtures (volume basis) of oct‐1‐en‐3‐ol and Hank's buffer into the assay buffer. We performed the study in Hank's buffer containing 5 mM of Ca^2+^ but noticed insignificant differences in the fluorescence emissions from each sample (Figure 9). Subsequent addition of VUAA1 to a final concentration of 1 mM in the solutions activated Orco expression by the PP‐Orco cultures equally (data not shown). These observations confirm that oct‐1‐en‐3‐ol does not interact with Orco of *A*.* gambiae*.

## 4 DISCUSSION

Most mosquito and insect repellants that are presently on the market were discovered using an apparatus known as an olfactometer. It is quintessentially a Y‐shaped chamber that studies the behavior of mosquitoes that are housed at the base of Y when they are exposed to odorant samples in both or either one of its two arms (Beavers et al., 1982). Not only are these experiments slow to perform, but olfactometers offer limited control over critical experimental parameters (DeGennaro et al., 2013; Kröber et al., 2010). The *Pichia* biosensor described in this study directly addresses these limitations. We have conclusively demonstrated that *P*.* pastoris* can functionally express Orco and that the level of expression can be modulated by varying the concentration of the transcriptional inducer. We have also identified culturing conditions that facilitate optimal expression of the protein. We then tested the biosensor by exposing it to VUAA1, one of the strongest agonists of Orco reported in the literature. Although it is known that Orco is a cationic channel, we observed that it functions like a TRP cationic channel after activation, even when expressed heterologously. Moreover, the fluorescent signal from the assay can be titrated by adjusting the concentration of either the stimulatory odorant or concentration of extracellular Ca^2+^ in the assay buffer. The EC50 s of VUAA1 for the *Pichia* expressed Orco were determined to be 0.83 mM and 0.41 mM when the Ca^2+^ concentrations in the assay buffer are 1 and 5 mM, respectively.

We also exposed the biosensor to citronella oil and oct‐1‐en‐3‐ol. Citronella oil is a widely used insect repellant, and oct‐1‐en‐3‐ol is a metabolite present in human sweat that is the primary attractant of mosquitoes. Previous studies have revealed that oct‐1‐en‐3‐ol does not interact with Orco (Carey et al., 2010). However, the effect of citronella oil on the protein is poorly understood. In *D*.* melanogaster*, citronellal, the primary constituent of citronella oil, is an agonist of Orco as well as TRPA1 receptors (Kwon et al., 2010). The *Pichia* biosensor corroborates that oct‐1‐en‐3‐ol does not interact with Orco of *A*.* gambiae*, but we determined that either citronellal or a minor constituent of citronella oil interferes with Orco. The ligand is either a negative allosteric modulator of Orco or an antagonist that is weakly competitive with VUAA1. We believe it is possible to further increase the sensitivity and signal‐to‐noise ratio of the biosensor by maintaining a higher concentration of Ca^2+^ in the assay buffer. *P*.* pastoris* has been shown to grow normally at extracellular Ca^2+^ concentrations as high as 100 mM (Miseta et al., 2002). We are also confident that the fluorescence emitted by the cells is a product of channel activation and not any other phenomena since the physiological concentration of Ca^2+^ ions in the cytoplasm of *P*.* pastoris* ranges between 50 and 200 nM (Cui et al., 2009; Miseta et al., 2002).

Similar biosensors have been constructed previously using HEK293 cells (Rinker et al., 2012), Sf9 cells (Jordan & Challiss, 2011; Kiely et al., 2007), and *Xenopus* oocytes (Misawa et al., 2010). Not only are these cell lines cumbersome and expensive to maintain, but they also require the use of patch clamping to assess receptor activation or deactivation, which is incompatible with high‐throughput screening. Among these competing platforms, only the *Xenopus* system has been adapted to a relatively high‐throughput microfluidics screening platform. However, the transformation of *Xenopus* oocytes is slow and has low efficiency. In contrast, not only is the *Pichia* biosensor comparably sensitive as these systems (Butterwick et al., 2018; Chen & Luetje, 2012), but it is also easier to maintain and deploy and simpler to modify and optimize. *P*.* pastoris* also has a faster doubling time (Panagiotou et al., 2011) and does not require the use of high doses of antibiotics, which is a significant advantage over other screening platforms (Fleischer et al., 2018; Jones et al., 2011; Rinker et al., 2012). Moreover, since all 79 ORs and Orco of female *A*.* gambiae* mosquitoes exhibit a high degree of structural and topological similarity within the membrane, the system is highly modular and can be used to investigate any of these proteins by co‐expressing them with Orco.

## 5 CONCLUSION

The *Pichia* biosensor developed in this study is sensitive and could form the basis of miniaturized, high‐throughput, and precise assays for identifying chemicals that can interact with mosquito olfactory proteins. Nevertheless, it should be noted that biosensors such as the type developed in this study can only probe receptor‐ligand interactions. Behavioral experiments using live mosquitoes are still needed to validate the hits identified using the biosensor. Whole‐cell biosensors such as the one described herein can winnow down and focus the chemical search space for ensuing behavioral experiments using live mosquitoes. Additionally, whole‐cell biosensors can also accelerate medicinal chemistry, which could then facilitate systematic elucidation of structure–activity relationships and the subsequent identification of effective repellants through lead optimization (Jones et al., 2011). Beyond repellant screening, biosensors *c*ould also be used as an investigative tool in other fields such as entomology, agriculture, and aromachology.

## AUTHOR CONTRIBUTIONS

**Julia Nogueira Varela:** Conceptualization (equal); Data curation (equal); Formal analysis (equal); Investigation (equal); Methodology (equal); Writing‐original draft (equal). **Vikramaditya Ganapati Yadav:** Conceptualization (equal); Data curation (equal); Formal analysis (equal); Funding acquisition (lead); Investigation (equal); Methodology (equal); Project administration (lead); Supervision (lead); Visualization (lead); Writing‐original draft (equal); Writing‐review & editing (lead).

## CONFLICTS OF INTEREST

None declared.

## ETHICS STATEMENT

None required.

## Data Availability

All data except for fluorescence recordings have been provided in their unprocessed form in the results section of this paper. The fluorescence data presented in the paper are normalized readings, and the normalization protocol has been described in the methods section.
